# Binding of the 9-*O*-*N*-aryl/arylalkyl Amino Carbonyl Methyl Substituted Berberine Analogs to tRNA^phe^

**DOI:** 10.1371/journal.pone.0058279

**Published:** 2013-03-19

**Authors:** Anirban Basu, Parasuraman Jaisankar, Gopinatha Suresh Kumar

**Affiliations:** 1 Chemistry Division, CSIR-Indian Institute of Chemical Biology, Kolkata, India; 2 Biophysical Chemistry Laboratory, CSIR-Indian Institute of Chemical Biology, Kolkata, India; University of South Florida College of Medicine, United States of America

## Abstract

**Background:**

Three new analogs of berberine with aryl/arylalkyl amino carbonyl methyl substituent at the 9-position of the isoquinoline chromophore along with berberrubine were studied for their binding to tRNA^phe^ by wide variety of biophysical techniques like spectrophotometry, spectrofluorimetry, circular dichroism, thermal melting, viscosity and isothermal titration calorimetry.

**Methodology/Principal Findings:**

Scatchard binding isotherms revealed that the cooperative binding mode of berberine was propagated in the analogs also. Thermal melting studies showed that all the 9-*O*-*N*-aryl/arylalkyl amino carbonyl methyl substituted berberine analogs stabilized the tRNA^phe^ more in comparison to berberine. Circular dichroism studies showed that these analogs perturbed the structure of tRNA^phe^ more in comparison to berberine. Ferrocyanide quenching studies and viscosity results proved the intercalative binding mode of these analogs into the helical organization of tRNA^phe^. The binding was entropy driven for the analogs in sharp contrast to the enthalpy driven binding of berberine. The introduction of the aryl/arylalkyl amino carbonyl methyl substituent at the 9-position thus switched the enthalpy driven binding of berberine to entropy dominated binding. Salt and temperature dependent calorimetric studies established the involvement of multiple weak noncovalent interactions in the binding process.

**Conclusions/Significance:**

The results showed that 9-*O*-*N*-aryl/arylalkyl amino carbonyl methyl substituted berberine analogs exhibited almost ten folds higher binding affinity to tRNA^phe^ compared to berberine whereas the binding of berberrubine was dramatically reduced by about twenty fold in comparison to berberine. The spacer length of the substitution at the 9-position of the isoquinoline chromophore appears to be critical in modulating the binding affinities towards tRNA^phe^.

## Introduction

**Figure 1:**
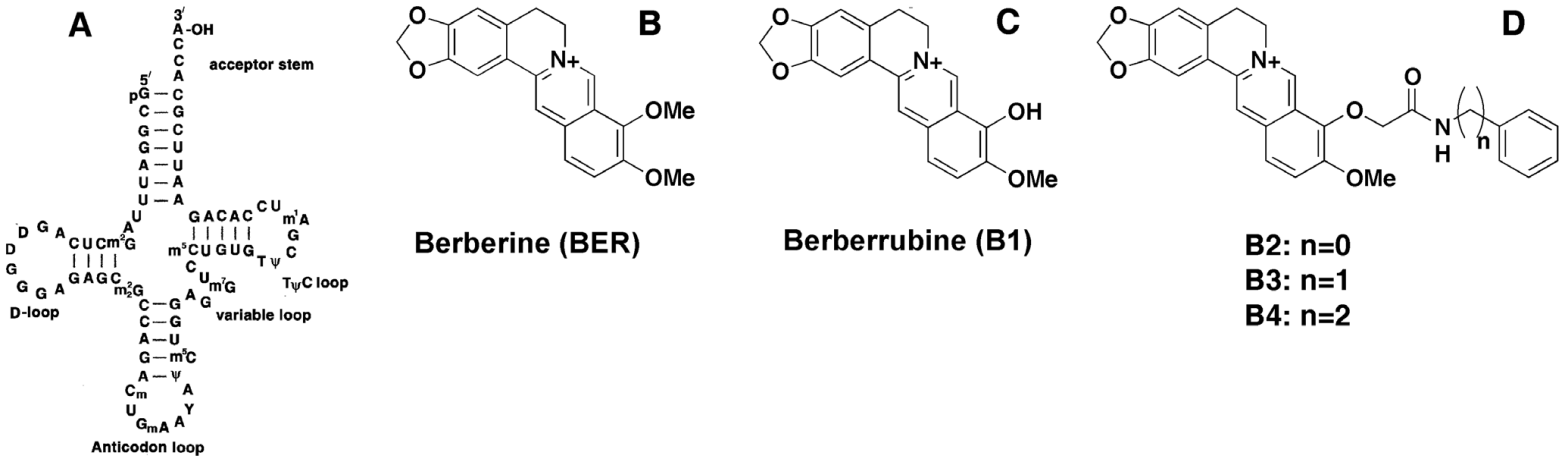
Chemical structures. (A) cloverleaf structure of tRNA, (B) berberine, (C) berberrubine and (D) analogs B2, B3, B4.

There has been a remarkable increase in our understanding of the critical roles played by RNA in a variety of structural, regulatory and enzymatic processes in the cells [1]. The discovery of a number of microRNAs and understanding of their functions have led to further interest for targeting RNA as a molecule for therapeutic intervention [2], [3]. Furthermore, the emerging knowledge of the role of cellular RNA molecules in many diseases like HIV AIDS, hepatitis C and viral infections has also led to immense interest in developing RNA-targeted therapeutic agents [4]–[7]. Transfer RNAs play a pivotal role in protein synthesis and translation. Translation is carried out by tRNA^phe^ (hereafter tRNA) through the relationship between its anticodon and the associated amino acid. tRNA is the small RNA chain consisting of 73–93 nucleotides transferring a specific amino acid to a growing polypeptide chain at the ribosomal site of protein synthesis during translation. It has a cloverleaf structure (Fig. 1A) that shows a high degree of folding stabilized by base stacking, base pairing and other tertiary interactions. It has a 3′ terminal site for amino acid attachment which is catalyzed by an aminoacyl tRNA. The structure of tRNA contains a three base region called anticodon that can base pair to the codon region on mRNA. tRNA is one of the fairly well characterized structure and the cloverleaf structure of tRNA may represent unique sites for small molecule binding and recognition. A rational design of RNA targeted small molecule therapeutics essentially requires both detailed knowledge of the structural aspects of RNAs and the molecular nature of the interaction phenomena. Detailed RNA-small molecule binding studies have been greatly hampered due to the lack of high-resolution conformational information and the highly complex RNA structures. However, progress in structural aspects of RNAs through X-ray, NMR and computational studies have enabled detailed studies on the elucidation of the mode and mechanism of interaction of many new RNA binding molecules [7]–[9]. Our laboratory has been studying the interaction of a group of natural isoquinoline alkaloids with RNA targets like tRNA and poly(A) [10]–[23]. From these studies it was revealed that natural isoquinoline alkaloids are useful in selectively targeting RNA through various means [10]–[25]. It is well known that the RNA molecules have highly versatile structures that can fold into a multitude of conformations, and these complex structural motifs may serve as potential binding pockets for specific drug recognition sites that need to be understood in details. It would be interesting to take advantage of these promising recognition capabilities of RNAs to develop new RNA binders as modulators of cellular functions [5], [26], [27].

Natural products owing to their large chemical diversity and pharmacological activity have been widely accepted as potential high quality pools in drug screening studies. Berberine (Fig. 1B), is one such naturally occurring isoquinoline alkaloid that is endowed with myriad therapeutic potential. The strong antineoplastic effect of berberine appears to suppress the growth of a wide variety of tumor cells [28], [29]. In recent years pharmacological activities of this alkaloid as antihypertensive, antiinflammatory, hepatoprotective, antioxidant, antidepressant, antidiarrhoeal and as antimicrobial agent have been amply demonstrated [30]–[36]. However, DNA and RNA binding affinities of berberine are only moderate and hence appropriate structural modifications are essential for application as therapeutic agent with high binding affinity and specificity. The importance of 9-position substitutions in berberine's pharmacological activity is well documented [37]–[41]. Analogs of berberine with substitutions at the 9-position have been shown to possess better anticancer activity in human cancer cells, enhanced DNA and RNA binding and possess higher topoisomerase poisoning activity and telomerase inhibition properties [21], [22], [37]–[39], [41]–[47]. We have recently studied the binding of a series of novel 9-*O*-*N*-aryl/arylalkyl amino carbonyl methyl substituted berberine analogs to double stranded DNA and poly(A) and showed that they bound to duplex DNA and single stranded poly(A) exhibiting remarkably enhanced binding affinities. However, the binding of the 9-substituted analogs to tRNA have not been yet studied. tRNA represents one of the most common and most thoroughly characterized natural RNAs. They constitute about 15% of the total cellular RNA and bind to the A-site of ribosomes. Recently there has been an increasing interest in understanding the binding of many small molecules to tRNA [48]–[53]. Here we present the results of our investigation on the binding of berberrubine (Fig. 1C) and 9-*O*-*N*-aryl/arylalkyl amino carbonyl methyl substituted berberine analogs (Fig. 1D) to tRNA and compare it with the tRNA–berberine complexation from multifaceted biophysical experiments.

## Materials and Methods

### Biochemicals

tRNA^phe^ (yeast) and berberine were purchased from Sigma-Aldrich Corporation (St. Louis, MO, USA) and were used as received. The ratio of the absorbance at 260 to 280 nm for tRNA was around 1.85 indicating that the sample was free from protein contaminations. Analogs B1, B2, B3 and B4 (Fig. 1C, D) were synthesized, purified and characterized as reported earlier [47], [54].

### Preparation of Stock Solutions

tRNA solution was prepared in the experimental buffer and dialyzed in cold room. Its concentration was determined spectrophotometrically using a molar extinction coefficient (ε) of 6,900 (M^−1^ cm^−1^) at 260 nm expressed in terms of nucleotide phosphates [11], [12]. The solutions of berberine, B1, B2, B3 and B4 were also prepared in the experimental buffer and their concentrations were determined by applying the molar extinction coefficient (ε) values of 22,500, 22,500, 25,000, 26,000 and 26,500 M^−1^ cm^−1^, respectively, at 345, 373, 345, 344 and 345 nm. No deviation from Beer's law was observed for the alkaloids in the concentration range employed in this study. All buffer salts and other reagents were of analytical grade or better. Solutions were freshly prepared in the buffer and kept protected in the dark. All experiments were conducted in filtered 10 mM citrate-phosphate (CP) buffer, pH 7.0, containing 5 mM of Na_2_HPO_4_ prepared in deionised and triple distilled water. pH was adjusted with citric acid.

### Absorbance Spectroscopy

The absorption spectra of the alkaloids mixed with or without RNA were obtained using a Jasco V660 unit (Jasco International Co, Hachioji, Japan) at 20±0.5°C in matched quartz cuvettes of 1 cm path length. Titrations were performed keeping a constant concentration of the alkaloid and varying the tRNA concentration following generally the methods described earlier [55]–[57].

### Fluorescence Spectroscopy

Steady state fluorescence measurements were performed on a Hitachi F4010 fluorescence spectrometer (Hitachi Ltd., Tokyo, Japan) or RF- 5301 PC fluorimeter (Shimadzu Corporation, Kyoto, Japan) in fluorescence free quartz cuvettes of 1 cm path length as described previously [56], [57] where a fixed concentration of analog was titrated with increasing concentrations of the tRNA. The excitation wavelength for the alkaloids was fixed at their respective absorption maxima. All fluorescence measurements were done keeping excitation and emission band passes of 5 nm. The sample temperature was maintained at 20±0.5°C using Eylea Uni Cool U55 water bath (Tokyo Rikakikai, Tokyo, Japan). Uncorrected fluorescence spectra are presented.

### Binding Affinity Calculations

In absorption spectroscopy, following each addition of the alkaloid to the RNA solution (50 µM), the total alkaloid concentration present was calculated as 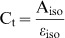 from the absorbance at the respective isosbestic point (A_iso_), where ε_iso_ is the molar extinction coefficient at the isosbestic point. The expected absorbance (A_exp_) at the wavelength maximum was calculated using the relation 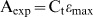, where ε_max_ is the molar extinction coefficient at the wavelength maximum. The difference in A_exp_ and the observed absorbance (A_obsd_) was then used to calculate the amount of bound alkaloid as 
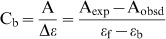
. The concentration of the free alkaloid was determined by the difference 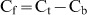. The extinction coefficient of the completely bound alkaloid was determined by adding a known quantity of the alkaloid to a large excess of RNA and on the assumption of total binding, 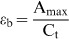. Alternatively, the absorbance of a known quantity of the alkaloid was monitored at the wavelength maximum while adding known amounts of RNA until no further change in absorbance was observed. Both of these methods gave identical values within experimental error. In fluorescence, C_b_ was calculated from the relation 
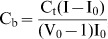
, where C_t_ is the total alkaloid concentration, I is the observed fluorescence intensity, I_o_ is the fluorescence intensity of identical concentration of the alkaloid in the absence of RNA, and V_o_ is the experimentally determined ratio of the fluorescence intensity of totally bound alkaloid to that of the free alkaloid. Free alkaloid concentrations (C_f_) were obtained from the relationship 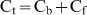. The binding ratio r is defined as 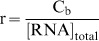. Binding data obtained from spectrophotometric and spectrofluorimetric titration was cast into Scatchard plot of r/C_f_ versus r. The Scatchard isotherms with positive slope at low r values were analyzed using the following McGhee-von Hippel equation for cooperative binding [57]–[59].
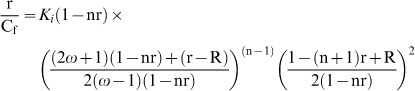
(1)where,
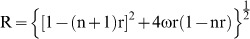
Here, *K_i_* is the intrinsic binding constant to an isolated binding site, n is the number of base pairs excluded by the binding of a single alkaloid molecule, and ω is the cooperativity factor. All the binding data were analyzed using the Origin 7.0 software (Origin Laboratories, Northampton, MA, USA) that determines the best-fit parameters of *Ki*, n, and ω to eq 1.

The spectral data were also analyzed by constructing Benesi-Hildebrand plots [60] using the following relation,
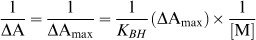
(2)where ΔA is the change in absorbance at a given wavelength and [M] is the concentration of tRNA. By plotting the reciprocal of the absorbance intensity with respect to reciprocal concentration of tRNA, the Benesi-Hildebrand association constant for the complex formation (*K_BH_*) was calculated from the ratio of the slope to the intercept [60], [61].

### Continuous Variation Analysis (Job plot)

Continuous variation method of Job [62]–[64] was employed to determine the binding stoichiometry in each case using fluorescence spectroscopy. At constant temperature, the fluorescence signal was recorded for solutions where the concentrations of both RNA and alkaloids were varied keeping the sum of their concentrations constant. The difference in fluorescence intensity (ΔF) of the analogs in the absence and presence of RNA was plotted as a function of the input mol fraction of each alkaloid as reported previously [11], [64]. Break point in the resulting plot corresponds to the mol fraction of the bound alakloid in the complex. The stoichiometry was obtained in terms of RNA-alkaloid [(1-χ_alkaloid_)/χ_alkaloid_] where χ_alkaloid_ denotes the mol fraction of the respective alkaloid. The results reported are average of at least three experiments.

### Fluorescence Quenching

Fluorescence quenching experiments were performed by mixing, in different ratios, two solutions, viz. KCl and K_4_[Fe(CN)_6_], at a fixed total ionic strength. At a constant P/D (RNA/alkaloid molar ratio) fluorescence intensity was monitored as a function of varying concentration of the ferrocyanide as described previously [13], [65]. The data were plotted as Stern-Volmer plots of relative fluorescence intensity (F_o_/F) versus [Fe(CN6]^4−^ concentration according to the Stern-Volmer equation, 
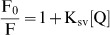
, where F_o_ and F denote the fluorescence emission intensities in the absence and presence of [Fe(CN)_6_]^4−^ and [Q] is the quencher concentration. *K_sv_* is the Stern-Volmer quenching constant, which gives an estimate of the efficiency of quenching by the quencher.

### Viscosity measurements

The viscosity of the tRNA–alkaloid complexes was determined by measuring the time taken to flow through a Cannon-Manning semi micro size 75 capillary viscometer (Cannon Instruments Company, State College, PA, USA). The viscometer was submerged vertically in a thermostated bath maintained at 20±1°C. The experimental protocol for the measurement of flow times and the calculation of relative viscosities were as reported previously [11].

### Quantum Efficiency Measurements

Fluorescence quantum efficiency was calculated using the following equation [66]

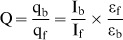
(3)where ε_f_ and ε_b_ represent the molar extinction coefficients of the free and bound alkaloids and I_b_ and I_f_ are the fluorescence intensities of bound and free alkaloid, respectively. The quantum efficiency was determined according to the procedure, described earlier [67].

### Optical Melting Studies

Absorbance versus temperature curves (melting profiles) of RNA and RNA-alkaloid complexes were measured on the Shimadzu Pharmaspec 1700 unit (Shimadzu Corporation) equipped with the peltier controlled TMSPC-8 model accessory as described earlier [11], [68]. In a typical experiment, the RNA sample (50 µM) was mixed with the varying concentrations of the alkaloid under study and heated at a rate of 0.5°C/min. monitoring continuously the absorbance change at 260 nm. The *T_m_* is the midpoint of the melting transition as determined by the maxima of the first derivative plots.

### Circular Dichroism Measurements

A JASCO J815 spectropolarimeter interfaced with a thermal programmer (Jasco model 425L/15) and controlled by a PC was used for all circular dichroism measurements at 20±0.5°C as reported earlier [13], [46], [57]. A rectangular strain free quartz cuvette of 1 cm path length was used. A scan rate of 50 nm min^−1^, a bandwidth of 1.0 nm and a sensitivity of 100 millidegrees was set for the measurements. Each spectrum was averaged from five successive accumulations and was base line corrected and smoothed within permissible limits using the Jasco software. The molar ellipticity [θ] values are expressed in terms of nucleotide phosphate concentration in the wavelength region of 220–400 nm. The calibration of the CD unit was routinely checked using aqueous solution of d-10 ammonium camphor sulphonate.

### Isothermal Titration Calorimetry

Isothermal titration calorimetry (ITC) experiments were performed on a VP ITC unit (MicroCal, Northamption, USA). Titrations were performed at four temperatures viz. 283.15, 288.15, 293.15 and 298.15 K as described earlier [53]. tRNA and alkaloid solutions were degassed on the MicroCal's Thermovac unit before loading to avoid the formation of bubbles in the calorimeter cell. The instrument control, titration and analysis were performed using the dedicated Origin 7.0 software provided with the unit. Titrations were performed by injecting the alkaloid solution from the rotating syringe into the RNA solution kept in the calorimeter cell. The duration of each injection was 10 seconds and the delay time between each injection was 240 seconds. The initial delay before the first injection was 60 seconds. Corresponding control experiments to determine the heat of dilution of the alkaloid were performed by injecting identical volumes of the same concentration of the alkaloid into the buffer. The heat of dilution of injecting the buffer into the RNA solution was measured and was found to be negligible. Each injection generated a heat burst curve (micro calorie per second versus time). The resulting thermograms after appropriate control corrections were analyzed using single set of binding sites model of Levenberg-Marquardt non-linear least squares curve fitting algorithm, inbuilt with the software of the unit. The association constant *K*_a_, stoichiometry N, and molar heat of binding Δ*H* ^0^ were obtained using the following relation:
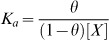
(4)where θ = fraction of sites occupied by drug *X*, and [*X*] = concentration of free drug. Therefore, the total concentration of drug (free and bound), X_t_ is given by
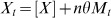
(5)where M_t_ is the bulk concentration of macromolecule in the active cell volume V_cell_. The total heat content Q of the solution in the active cell volume is
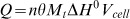
(6)


Considering the volume change ΔV_i_ accompanying the injection i, the heat released, ΔQ_i_ from the i^th^ injection is
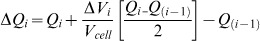
(7)


Binding free energy and entropy change were obtained using the relation
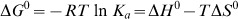
(8)where *R* signifies the universal gas constant (1.9872 cal K^−1^ mol^−1^). Specific heat capacity changes (Δ*C_p_^o^*) were derived from a plot of enthalpy (Δ*H^0^*), versus temperature (T), at constant pressure, using the following relation
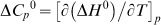
(9)


The ITC unit was periodically calibrated electrically and verified with water-water and water-methanol dilution experiments as per the criteria of the manufacturer.

## Results and Discussion

### Absorption Spectral Studies

**Figure 2:**
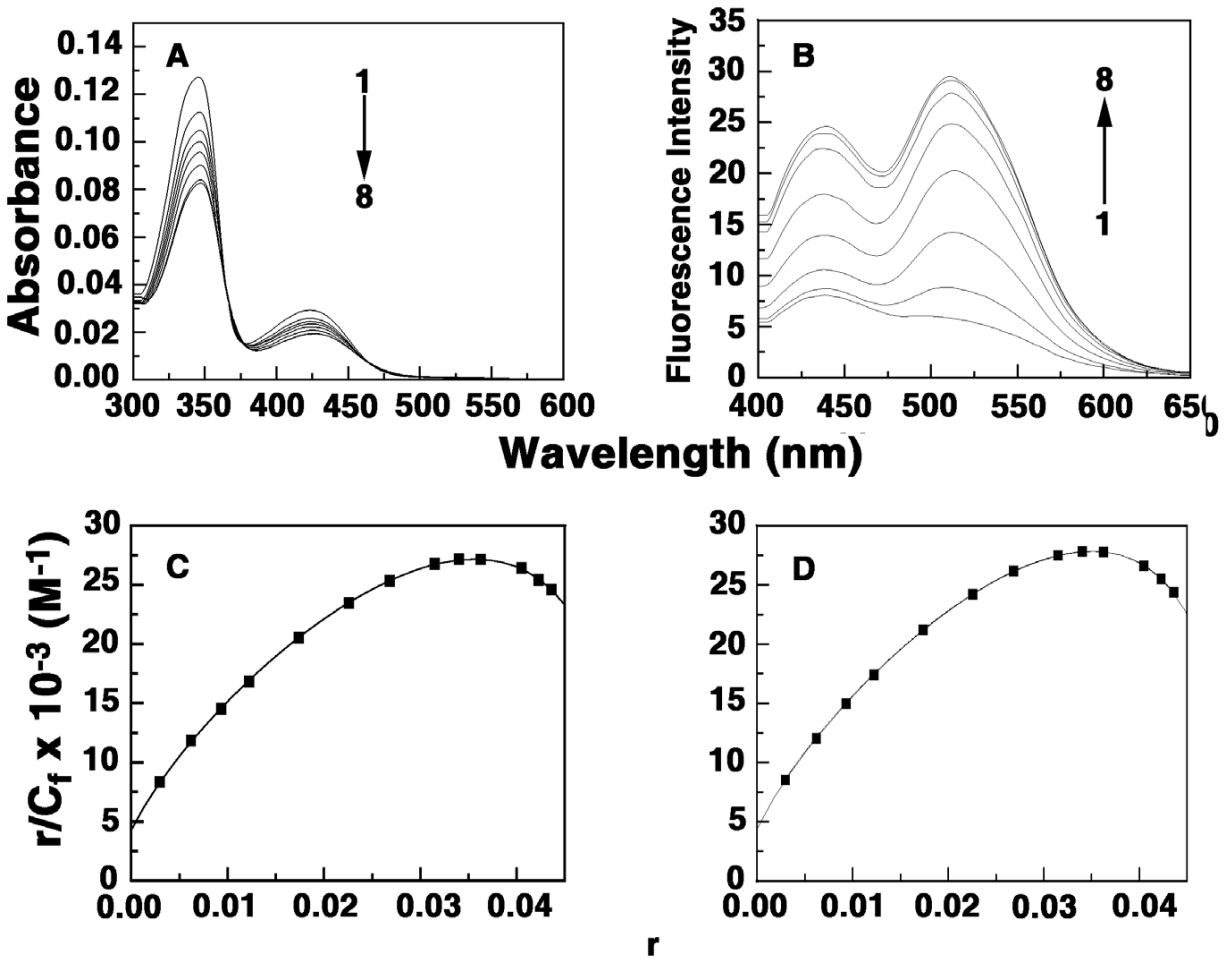
Absorption and fluorescence spectral studies of the analogs with tRNA. (A) Absorption spectral changes of analog B4 (curve 1) with increasing concentration of tRNA (curves 2–8), (B) fluorescence spectral changes of analog B4 (curve 1) with increasing concentration of tRNA (curves 2–8) (C) Scatchard plot for analog B4-tRNA complexation from absorbance and (D) Scatchard plot for analog B4-tRNA complexation from fluorescence.

BER and its 9-*O*-*N*-aryl/arylalkyl amino carbonyl methyl substituted analogs have characteristic absorption spectra in the 300–600 nm region with intense peak around 345 and 420 nms that provide a convenient handle to monitor the RNA binding. Pronounced hypochromic and bathochromic effects were observed in this spectral region when mixed with increasing concentrations of tRNA, revealing strong intermolecular association. Such spectral changes have been usually interpreted to arise from a strong interaction between the π electron cloud of the interacting small molecule and the nucleic acid bases presumably due to intercalation [11]. The presence of three sharp well defined isosbestic points in each case enabled the assumption of a two state system consisting of bound and free alkaloid at any particular wavelength. In Fig. 2A absorption spectral changes in analog B4 on titration with increasing concentration of tRNA are presented. However, analog B1 exhibited absorption maxima at 328, 373 and 482 nm and in presence of increasing concentration of tRNA hypochromic changes were observed in its absorption spectra (not shown), but the extent of hypochromicity was significantly lower compared to BER and its 9-*O*-*N*-aryl/arylalkyl amino carbonyl methyl substituted analogs. Unlike BER and its analogs B2-4 no isosbestic points were observed for analog B1 in presence of tRNA. This indicated *prima facie* weaker binding of the analog B1 compared to BER and its other analogs.

### Fluorescence Titration Studies

Berberine is a weak fluorophore with an emission maximum around 444 nm when excited at 345 nm [69]. The 9-*O*-*N*-aryl/arylalkyl amino carbonyl methyl substituted analogs are comparatively more fluorescent with emission maxima in the range 440–455 nm when excited at 345 nm. Complex formation was monitored by titration studies keeping a constant concentration of the alkaloid and increasing the concentration of tRNA until saturation was achieved. Figure 2B shows the results from spectrofluorimetric titration of analog B4 with tRNA. In presence of increasing concentrations of tRNA pronounced enhancement in the fluorescence intensity of the analoge was observed with concomitant development of a peak in the 510–520 nm region. The remarkable enhancement of intensity with the analogs may be due to the stronger binding that leads to a decrease of the collisional frequency of the solvent molecules. Large fluorescence change is indicative of strong association resulting from an effective overlap of the electronic cloud of the bound analogs with the bases of tRNA. Furthermore, this also proposes the presence of the bound molecules to be in a more hydrophobic environment similar to an intercalated state compared to the free ones [11]. Analog B1 when excited at 373 nm exhibited emission maximum at 466 nm. In presence of tRNA only marginal changes were observed in its emission spectrum and no prominent peak was seen in the 520 nm region. This revealed that analog B1 has very weak binding affinity to tRNA.

### Elucidation of the Binding Affinity

The data obtained from spectrophotometric and spectrofluorimetric titrations were also analyzed by constructing Scatchard plots and then subjected to analysis by appropriate McGhee and von Hippel model. A representative Scatchard plot for analog B4-tRNA complexation is presented in Fig. 2C. The binding isotherm of BER and its analogs B2-4 with the tRNA had positive slope at low r values, indicating cooperativity of binding. Therefore, the cooperative binding mode of the parent alkaloid berberine was propagated in the analogs also [12]. The values of the overall binding affinity (*K*) which is a product of the cooperative binding affinity (*K*_i_) and the cooperativity factor (ω) are presented in Table 1. For the analog B4, the overall binding affinity (*K*_i_ω) was 9.22×10^5^ M^−1^ while for B2 and B3 the values were slightly lower and were 4.34×10^5^ and 6.40×10^5^ M^−1^, respectively. It can be observed that the magnitude of the binding affinity varied as B4>B3>B2>BER. However, the binding affinity value for analog B1 could not be evaluated by the Scatchard cum McGhee and von Hippel methodology because of the lack of isosbestic point in the titration with tRNA.

**Table 1:** Binding parameters of complexation of BER and its analogs with tRNA from spectrophotometric and spectrofluorimetric studies^a^.

	Absorbance		Fluorescence	
	Scatchard plot	Benesi-Hildebrand plot	Scatchard plot	Benesi-Hildebrand plot
Alkaloids	*K_i_*ω/10^5^ M^−1^	*K_BH_*/10^5^ M^−1^	*K_i_*ω/10^5^ M^−1^	*K_BH_*/10^5^ M^−1^
BER	1.69	1.14	1.77	1.22
B1	nd	0.043	nd	0.042
B2	4.34	4.25	4.27	4.04
B3	6.40	6.13	6.58	6.44
B4	9.22	8.74	9.41	8.89

a Average of four determinations. Binding constants (*K_i_* and *K_BH_*) were measured in citrate-phosphate buffer of 10 mM [Na^+^], pH 7.1 at 20°C. ω is the cooperativity factor. nd is not determinable.

The Scatchard plots constructed from fluorescence titration data also revealed cooperative binding for the analogs. A representative Scatchard plot for the binding of B4 to tRNA is presented in Fig. 2D. The overall binding constants as per the Scatchard analysis using the cooperative binding model of McGhee-von Hippel yielded binding affinity values of 4.27×10^5^ M^−1^ for B2, 6.58×10^5^ M^−1^ for B3 and 9.41×10^5^ M^−1^ for B4 against a value of 1.77×10^5^ M^−1^ for BER (Table 1). Therefore, a remarkable enhancement in the binding affinity was observed on introduction of the 9-*O*-*N*-aryl/arylalkyl amino carbonyl methyl substituent.

We also analyzed the spectral change data by the Benesi-Hildebrand protocol [60], [61]. The ratio of the intercept to the slope gave *K_BH_*, the apparent binding constant. From this analysis of the spectrophotometric data, it was found that the binding affinity values of BER and its analogs B1-4 to tRNA were 1.14×10^5^, 4.30×10^3^, 4.25×10^5^, 6.13×10^5^ and 8.74×10^5^ M^−1^, respectively. Similarly, from analysis of the spectrofluorimetric data the *K_BH_* values were deduced to be 1.22×10^5^, 4.20×10^3^, 4.04×10^5^, 6.44×10^5^ and 8.89×10^5^ M^−1^, respectively. These values are presented in Table 1. The values of the affinity obtained from this analysis were in excellent agreement with those deduced from McGhee-von Hippel analysis of the Scatchard plots.

### Determination of Quantum Efficiency

The quantum efficiency (Q) of a nucleic acid binding alkaloid is a measure of the energy transferred from the nucleic acid to the alkaloid upon complexation and is evaluated from the ratio of the quantum efficiency of the alkaloid bound to nucleic acid (q_b_) to the quantum efficiency of the free alkaloid (q_f_). Determination of quantum efficiency (Q) further supports the strong binding of the analogs to tRNA. A plot of ΔAbs. against the inverse of tRNA concentration gave an exponential plot (not shown) from which a quantum efficiency values of 3.99, 6.13 and 6.51, respectively, have been determined for analogs B2-4, which indicated enhancement of the energy of the bound alkaloid. Q>1 is indicative of enhancement of fluorescence intensity and greater retention of fluorescence energy by the bound analogs due to shielding within the binding site from quenching by the solvent [66].

### Binding Stoichiometry (Job plot)

**Figure 3:**
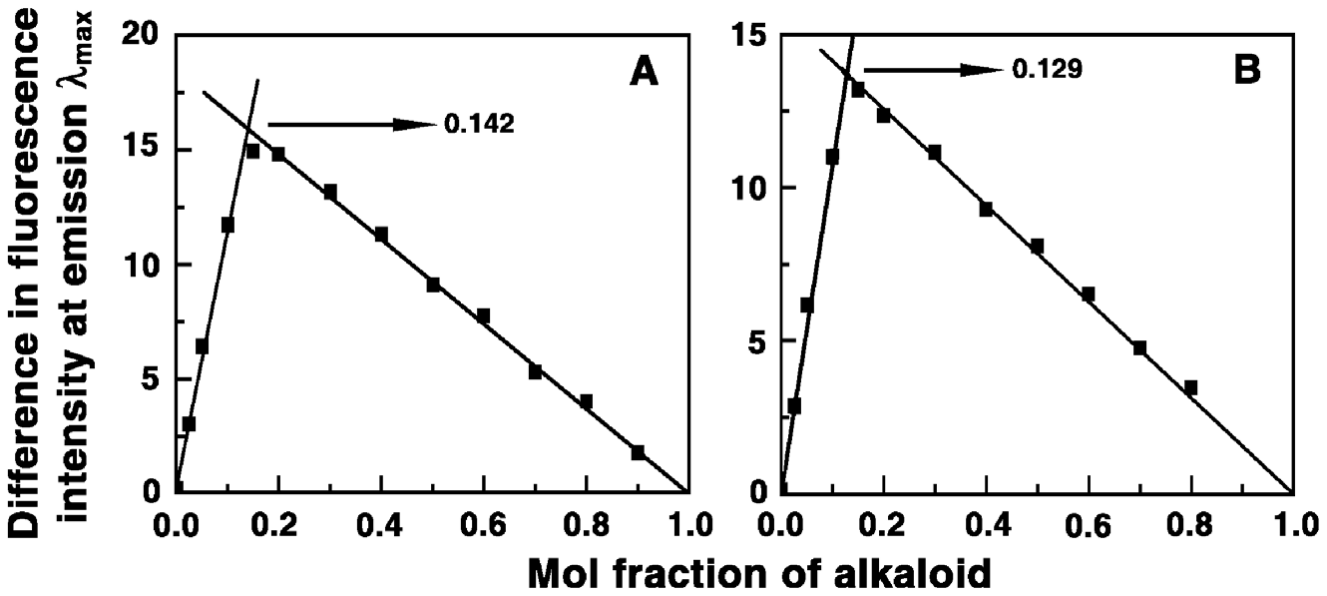
Job plot for the binding of the analogs with tRNA. (A) analog B3 and (B) analog B4.

To establish the binding stoichiometry of BER and its analogs with tRNA, continuous variation analysis procedure (Job plot) was performed in fluorescence at constant temperature [62]–[64]. The fluorescence signal was recorded for mixture of solutions where the concentrations of both tRNA and alkaloid were varied keeping the sum of their concentrations constant. The difference in fluorescence intensity (ΔF) of the alkaloids, at their respective emission maxima, in the absence and presence of the tRNA was plotted as a function of the input mol fraction of the alkaloids. The representative Job plots for analogs B3 and B4 are presented in Fig. 3. The plots revealed single break point indicating a single binding mode in each case. The break points in the plots correspond to the mol fraction of the bound alkaloid in the complex. The intersection of least square fitted lines at χ_alkaloid_ = 0.115, 0.154, 0.142 and 0.129 corresponds to stoichiometry values (number of tRNA bases bound per alkaloid) of 7.70, 5.49, 6.04 and 6.75, respectively, for the complexation of BER, B2, B3 and B4.

### Fluorescence Quenching Studies of Alkaloid-tRNA Complexes

**Figure 4:**
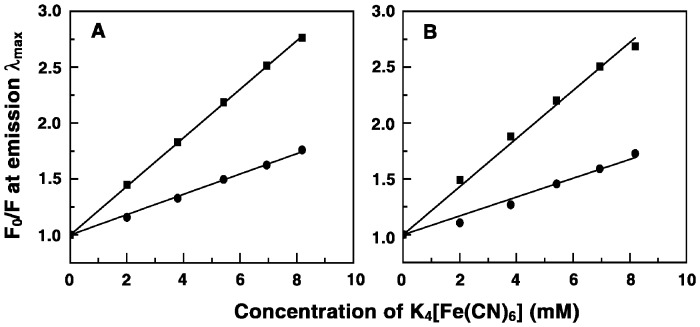
Stern-Volmer plots for the quenching of fluorescence by increasing concentration of [Fe(CN)_6_]^4−^. (A) analog B3 and (B) analog B4. Symbols are in the absence (▪) and in the presence (•) of tRNA.

[Fe(CN)_6_]^4−^ quenching experiment is a simple reliable method for investigating the binding mode of fluorescent small molecules [65]. Fluorescence quenching experiments were performed at a constant P/D ratio, monitoring the fluorescence intensity at emission maxima of alkaloid-tRNA complex as a function of changing concentration of K_4_[Fe(CN)_6_]. The anionic quencher would not be able to penetrate the negatively charged tRNA helix and if the alkaloid molecules are bound inside the RNA helix strongly by intercalation then little or no change in fluorescence is expected. Stern-Volmer plots clearly indicated that free molecules were quenched efficiently. More quenching was observed in case of BER and less quenching for the bound analogs B2, B3 and B4 indicating the bound analogs are indeed located in a relatively more protected environment than the parent alkaloid BER. However, for analog B1 more quenching was observed than BER indicating that B1 is exposed more to the anionic quencher compared to BER. The quenching constants (*K*_sv_) calculated were in the range 190–230 M^−1^ for the unbound BER and its analogs B1-4. For tRNA-bound BER and its analogs B1-4 the *K*_sv_ values were 121, 174, 102, 91 and 84 M^−1^, respectively. Stern-Volmer plots for the quenching of fluorescence intensity of B3 and B4 by increasing concentration of [Fe(CN)_6_]^4−^ in the absence and in the presence of tRNA is depicted in Fig. 4. The trend in the values varied as B4<B3<B2<BER<B1. This data suggests that the bound analog B1 is more exposed to the anionic quencher compared to BER whereas analogs B2-4 are located in a more protected environment compared to BER confirming stronger intercalative binding on tRNA.

### Viscosity studies

To further confirm the intercalative binding, viscosity measurements of tRNA–alkaloid complexes were performed. The viscosity of a solution of nucleic acid increases upon complexation with an intercalator due to the axial length enhancement making the RNA molecules more rigid on drug binding. Both these effects enhance the frictional coefficient resulting in viscosity increase. Although t-RNA is not a rod like molecule like DNA, we investigated whether the effect of binding of these analogs has any effect on its intrinsic viscosity. The results (Fig. S1) showed that the binding of all the alkaloids had profound effect on the intrinsic viscosity of tRNA and the net increase was larger and the change steeper with the analogs B2-4 compared to BER.

### Optical Melting Studies

Optical thermal melting of nucleic acid–alkaloid complexes is an important tool to investigate the interaction of small molecules [12], [47], [52]. External binding leading to the neutralization of the phosphate charges as well as the stacking interactions of intercalated molecules with the base pairs together contribute to the enhancement of the melting temperature (*T*_m_). In the absence of alkaloids, the melting of tRNA has three, not so well defined, transitions that showed transitions at 38.0, 50.0 and 63.0°C under the conditions of our experiment. This is in agreement to the data reported previously [12], [52]. The averaged melting temperature of the tRNA is about 50°C. Strong binding of small molecules may result in considerable enhancement of the melting temperature [12], [52]. In the presence of increasing concentrations of berberine the average *T*_m_ of tRNA increased from the initial 50°C by 4.5°C. Analogs B1-4 enhanced the T_m_ by 2.0, 6.5, 7.0, 8.0°C. At saturating concentrations, the average *T*_m_ of the tRNA was 54.5, 52.0, 56.5, 57.0 and 58.0°C, respectively, for BER and analogs B1-4 suggesting stronger binding of BER compared to analog B1 and remarkably stronger stabilization by analogs B2-4 compared to BER. Although increase in *T*_m_ may not directly be related with binding stability, the trend in the *T*_m_ values here certainly correlates well with the binding affinity values observed from other techniques.

### Circular Dichroism Spectral Studies

**Figure 5:**
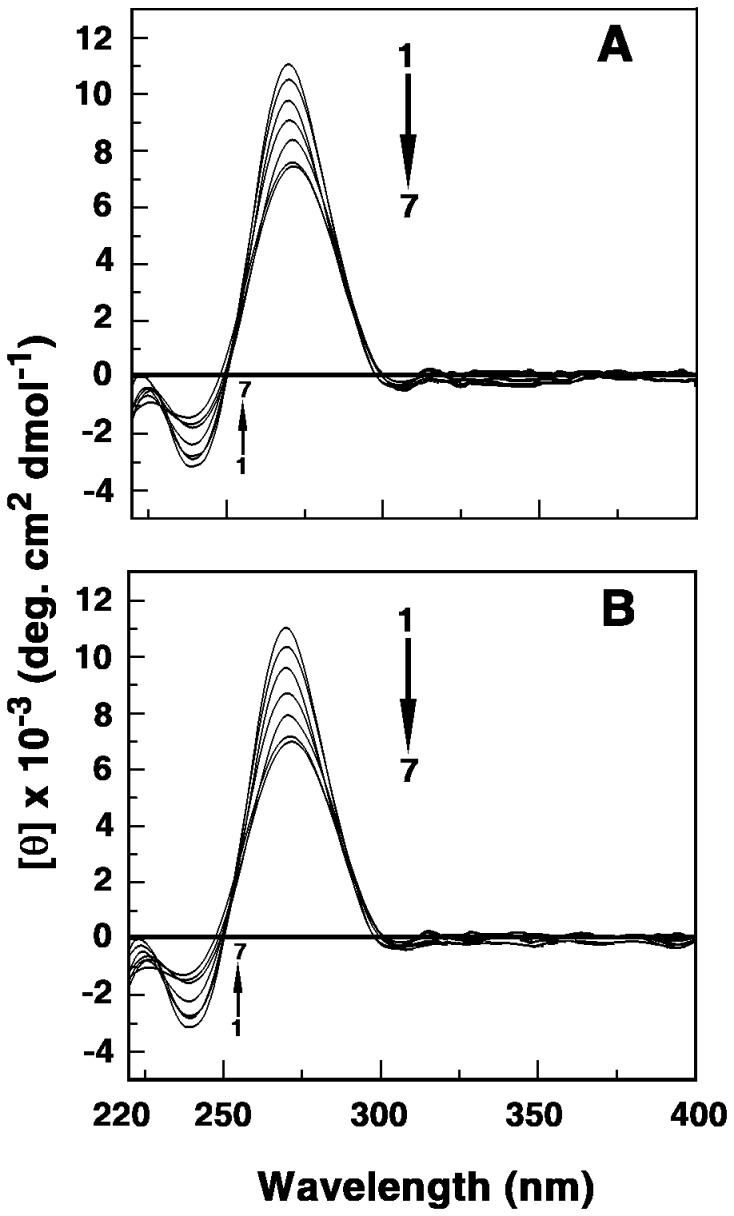
Circular dichroism spectra of the alkaloid-tRNA complexes. tRNA (50 µM) treated with increasing concentrations of analogs (A) B2 and (B) B4.

The CD spectrum of tRNA displays typical A-form conformation characterized by a large positive band in the 270 nm region and an adjacent weak negative band at 240 nm (spectrum 1 of Fig. 5). The alkaloid-induced changes in the RNA conformation (220–400 nm region) was recorded in the presence of varying D/P (alkaloid/RNA nucleotide phosphate molar ratio) values. In the presence of BER the ellipticity of the long wavelength positive band decreased as the interaction progressed with a small shift in the wavelength maximum till saturation was achieved. Similarly, the CD spectral changes accompanying the interaction of the berberine analogs B2-4 with tRNA was characterized by a remarkable decrease in the positive 270 nm peak indicating most likely a disruption of the base stacking interactions. Besides, comparatively less pronounced alterations in the negative CD band at 240 nm were also observed. For analog B1 the extent of decrease in the ellipticity of the positive band upon complexation with tRNA was significantly less compared to BER. Therefore the extent of decrease in the molar ellipticity of the positive 270 nm band followed the order B4>B3>B2>BER>B1. Similar trend was also reflected from other spectroscopic studies.

To gain further insights into the conformational aspects of the binding induced CD spectral measurements of the analogs in presence of tRNA was recorded the 300–700 nm region. However, no induced CD spectra with satisfactory signal to noise ratio were observed in the 300–700 nm region for these analogs.

### Thermodynamic Studies of Binding: Isothermal Titration Calorimetry

**Figure 6:**
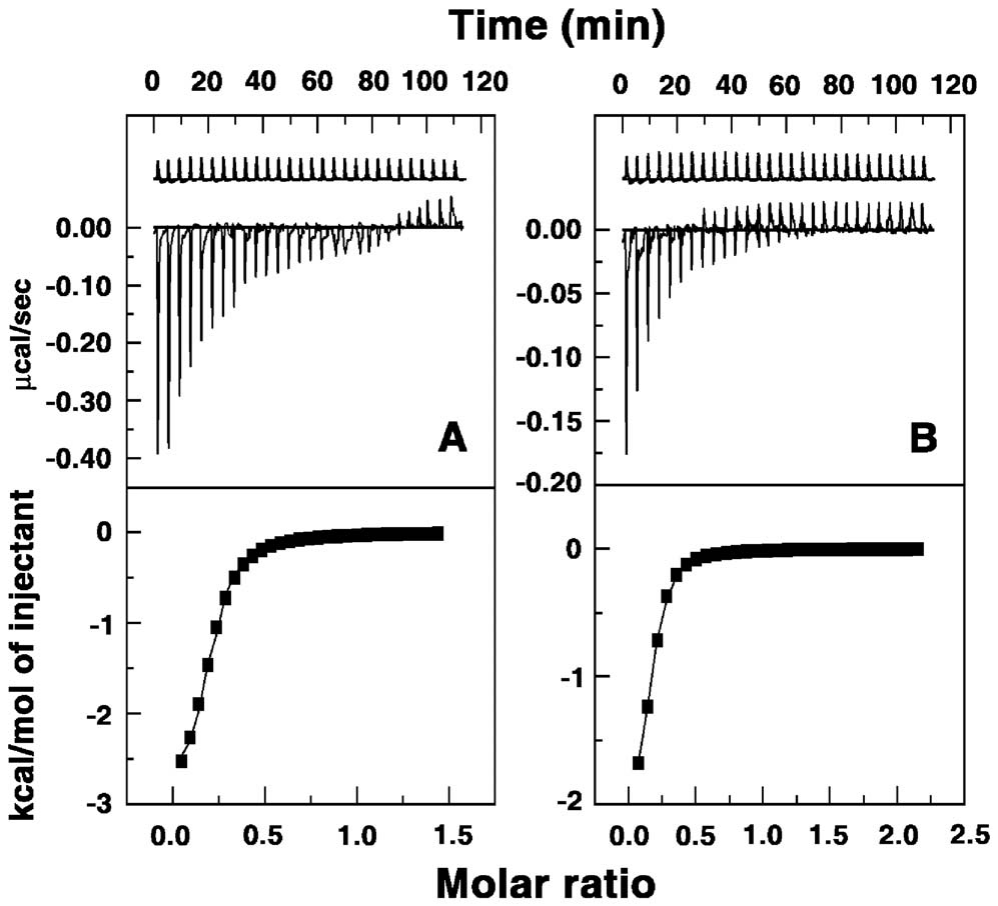
Representative ITC profiles for the complexation of the berberine analogs to tRNA. The profiles shown represent the sequential titration of successive aliquots of analogs (A) B2 and (B) B4 to tRNA (curve at the bottom), along with the dilution profiles (curves on the top offset for clarity). The top panel represents the raw data and the bottom panel shows the integrated heat data after correction of the heat of dilution. The symbols (▪) represent the data points that were fitted to a one-site model and the solid lines represent the best-fit data.

Isothermal titration calorimetry has become an effective tool to thermodynamically characterize the binding of small molecules to biomacromolecules [24], [25], [57]. The advantage of ITC is that it provided a complete thermodynamic profile of the binding such as Gibbs' energy change (Δ*G^0^*), enthalpy change (Δ*H^0^*) and entropy change (Δ*S^0^*) together with the affinity (*K_a_*) and stoichiometry (N) of the binding. Therefore, this technique was used to thermodynamically characterize the formation of tRNA–alkaloid complex. A representative calorimetric titration of analog B2 and B4 into the solution of tRNA at 20°C is presented in Fig. 6. Each injection heat burst curve in the figure corresponds to a single injection. These injection heats were corrected by subtracting the corresponding dilution heats derived from the titration of the alkaloid into buffer (Fig. 6, top panel, curves offset for clarity). In Fig. 6 (bottom panel) the resulting corrected heats are plotted against the respective molar ratio. In this panel the data points reflect the experimental data and the continuous line represents the calculated fits of the data to the binding model. The binding was characterized by exothermic heats. The corrected isotherms obtained at 20°C showed single site binding in all the cases indicating that one type of complexation is formed exclusively, thus enabling the fitting to a single site protocol. Fitting to one site model was also supported by the results from the Job plot analysis (vide supra). The results of the ITC experiments are presented in Table 2. The binding affinity values (*K_a_*) are of the order of 10^5^ M^−1^ for the analogs B2-4 which is remarkably higher in comparison to BER that had affinity of 8.6×10^4^ M^−1^. It is pertinent to note that analog B4 exhibited almost 10-fold higher binding affinity in comparison to BER. The interaction of the analog B1 with tRNA, the binding was negligible with only background heats. The binding affinity varied in the order B4>B3>B2>BER. The binding affinity values from ITC clearly suggested a drastic reduction in the affinity from BER to analog B1 and then a gradual increase from B2-4 compared to BER. This trend is similar to that observed from spectroscopic studies. The site size (n) values, which are the reciprocal of stoichiometry (N) values, in the range 5.15–6.37 also enhanced going from B2 to B4 and are in excellent agreement with the stoichiometry values obtained from Job plots. This enhancement in the site size values is probably indicative of the involvement of the side chain in the binding process. The Gibbs energy change for BER was about −6.61 kcal/mol while for analog B4 it was higher by 1.37 kcal/mol (in absolute values) at −7.98 kcal/mol. The Δ*G^0^* that significantly enhanced for the analogs B2-4 indicated enhanced binding preference of these analogs for tRNA. Therefore, the analoges side chain offered an additional module for strong contact and or better geometry with the tRNA bases and phosphate groups. Comparison of the thermodynamic parameters helps to elucidate the forces that govern the complexation. The ITC data showed an overall entropy driven binding for the analogs B2-4 in contrast to the enthalpy driven binding of BER. The large entropic contribution to the binding Gibbs energy observed for the binding of the analogs compared to BER may be interpreted in terms of binding-induced release of bound water and condensed sodium ions. With increase in the alkyl chain length of the 9-*O*-substitution the enthalpic contribution to the binding Gibbs energy gradually decreased while the entropic contribution to the binding Gibbs energy remarkably increased. The strongest binding was observed for B4, which was entropy driven (*T*Δ*S^0^* = 5.80 kcal/mol) with an enthalpy contribution of only −2.18 kcal/mol. Besides, the addition of the side chain at the 9-position enhanced the interaction free energy (in absolute values) by an additional 1.37 kcal/mol for B4 compared to BER, indicating more favorable contacts by the side chain on the tRNA bases. Thus, the addition of the *N*-aryl/arylalkyl amino carbonyl methyl side chain at the 9-position of the berberine chromophore significantly altered the energetics and binding affinity of berberine–tRNA interaction.

**Table 2:** Thermodynamic parameters for the association of alkaloids with tRNA from ITC^a^.

Alkaloids	*K_a_*/10^5^ M^−1^	N	Δ*H^0^* (kcal mol^−1^)	*T*Δ*S^0^* (kcal mol^−1^)	Δ*G^0^* (kcal mol^−1^)
BER	0.86±0.01	0.136±0.017	−3.97±0.03	2.64	−6.61±0.03
B2	4.67±0.03	0.194±0.012	−3.11±0.04	4.54	−7.65±0.04
B3	7.17±0.03	0.185±0.011	−2.80±0.02	5.04	−7.90±0.02
B4	8.20±0.04	0.157±0.009	−2.18±0.02	5.80	−7.98±0.02

a All the data in this table are derived from ITC experiments conducted in citrate-phosphate buffer of 10 mM [Na^+^], pH 7.0 and are average of four determinations. *K_a_* and Δ*H^0^* values were determined from ITC profiles fitting to Origin 7.0 software as described in the text. The values of Δ*G^0^* were determined using the equations and Δ*G^0^* = Δ*H^0^-T*Δ*S^0^*. N is the stoichiometry of binding. Analog B1 produced only background heat hence the data was not determinable. All the ITC profiles were fit to a model of single binding sites.

### Ionic Strength Dependence of the Binding and Parsing of the Gibbs Energy

**Figure 7:**
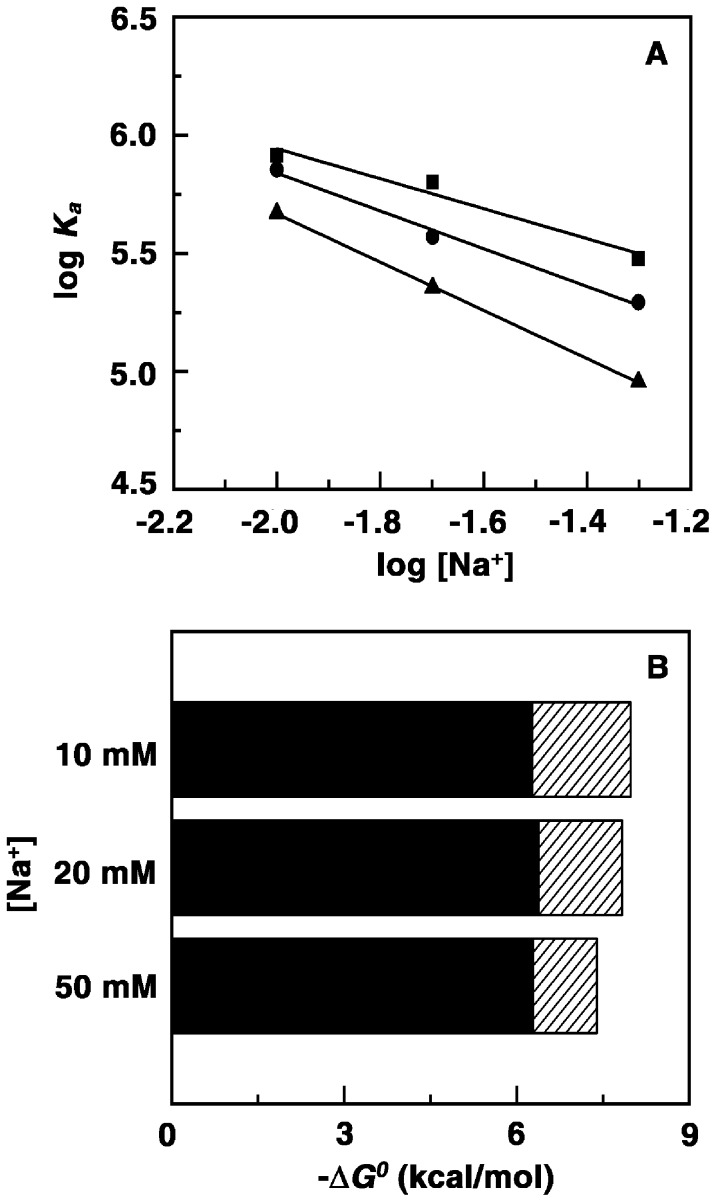
Plots of variation of salt dependent thermodynamic parameters. (A) Plot of log *K_a_* versus log [Na^+^] for the binding of analogs B2 (▪), B3 (•) and B4 (▴) to tRNA and (B) partitioned polyelectrolytic (Δ*G^0^_pe_*) (shaded) and nonpolyelectrolytic (Δ*G^0^_t_*) (black) contributions to the Gibbs energy of the B4-tRNA complexation at different [Na*^+^*] concentrations.

To provide an insight into the nature of the binding, the effect of salt concentration on the binding in ITC in conjunction with van't Hoff analysis was performed. All salt dependent studies have shown remarkable influence on the binding process. All the berberine analogs have the quaternary nitrogen atom that is positively charged at the 7-position of the isoquinoline chromophore. Therefore electrostatic interaction may be a significant driving force in the interaction of berberine analogs with nucleic acids. Around the tRNA cations are present as counter ions and charged ligands compete to expel these cations for phosphate neutralization; these are thermodynamically linked processes. In order to provide insights into the molecular details of such events ITC studies were performed at three different salt conditions viz. 10, 20 and 50 mM. The binding affinity reduced significantly when salt concentration was raised from 10 mM to 50 mM in each case. For analogs B2, B3 and B4 the *K_a_* values decreased from 4.67×10^5^ to 0.90×10^5^, 7.17×10^5^ to 1.96×10^5^ and 8.20×10^5^ to 3.00×10^5^ M^−1^, respectively. The Δ*G^0^* and Δ*H^0^* values also decreased concomitantly. Polyelectrolytic theories based on Manning's counter ions condensation model describe the process and provide a basis for interpreting this data [70]. From the polyelectrolytic theory, the slope of the best fit line for a plot of log *K_a_* versus log [Na^+^] is related to the counterion release by the following relation [71]

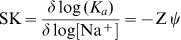
(10)where SK is equivalent to the number of counterions released upon binding of a small molecule, Z is the apparent charge of the bound ligand and ψ is the fraction of [Na^+^] bound per phosphate group. In Fig. 7A., the variation of log *K_a_* with log [Na^+^] for the binding of the analogs B2, B3 and B4 is presented. In each case the plot yielded straight line. The observed Gibbs energies of the interaction of the berberine analogs are in the range −6.68 to −7.98 kcal/mol (Table 3). In order to elucidate the dependence of *K_a_* on [Na^+^], the observed Gibbs energy can be partitioned between the polyelectrolytic (Δ*G^0^_pe)_* and non polyelectrolytic (Δ*G^0^_t_*) contributions (Fig. 7B) as per the following equation.
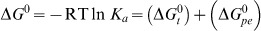
(11)


**Table 3:** Thermodynamic parameters for the association of alkaloids with tRNA from ITC at different salt concentrations^a^.

Alkaloids	[Na^+^] (mM)	*K_a_*/10^5^ M^−1^	Δ*H^0^* (kcal mol^−1^)	*T*Δ*S^0^* (kcalmol^−1^)	Δ*G^0^* (kcal mol^−1^)	Δ*G^0^_pe_* (kcal mol^−1^)	Δ*G^0^_t_* (kcalmol^−1^)
	10	4.67±0.03	−3.11±0.04	4.54	−7.65±0.04	−2.75	−4.90
B2	20	2.26±0.02	−2.86±0.04	4.36	−7.22±0.04	−2.34	−4.88
	50	0.90±0.01	−2.08±0.02	4.60	−6.68±0.02	−1.79	−4.89
	10	7.17±0.03	−2.80±0.04	5.04	−7.90±0.04	−2.16	−5.74
B3	20	3.71±0.02	−2.06±0.02	5.42	−7.51±0.02	−1.83	−5.68
	50	1.96±0.02	−1.97±0.02	5.13	−7.14±0.02	−1.40	−5.74
	10	8.20±0.04	−2.18±0.02	5.80	−7.98±0.02	−1.71	−6.27
B4	20	6.34±0.03	−1.98±0.02	5.85	−7.83±0.02	−1.45	−6.38
	50	3.00±0.02	−1.64±0.01	5.75	−7.39±0.01	−1.11	−6.28

a All the data in this table are derived from ITC experiments conducted in citrate-phosphate buffer of different [Na^+^], pH 7.0 and are average of four determinations. *K_a_* and Δ*H^0^* values were determined from ITC profiles fitting to Origin 7.0 software as described in the text. The values of Δ*G^0^* and *T*Δ*S^0^* were determined using the equations Δ*G^0^* = *−RT* ln*K_a_* and *T*Δ*S^0 = ^*Δ*H^0^* -Δ*G^0^.* Δ*G^0^_pe_* and Δ*G^0^_t_* are the polyelectrolytic and non polyelectrolytic contribution to Δ*G^0^*. All the ITC profiles were fit to a model of single binding.

The contribution to the Gibbs energy from the electrostatic interaction (polyelectrolytic) can be quantitatively determined from the relationship
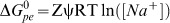
(12)where Zψ is the slope of the van't Hoff plot. At 50 mM [Na^+^] contribution to the Δ*G^0^_pe_* has been determined to be −1.11 kcal/mol for analog B4 (Table 3) which is about 15% of the total Gibbs energy change. At all salt concentrations, the Δ*G^0^_t_* that had large magnitude in each case remained almost invariant. Thus, although positively charged, the binding of these analogs is essentially dominated by hydrophobic forces and forces other than electrostatic. Therefore, the 9-*O*-*N*-aryl/arylalkyl amino carbonyl methyl side chain offers additional point for hydrophobic interaction with the tRNA bases and phosphates. This is similar to the results observed for berberine, palmatine and coralyne binding to ds DNA and RNAs where majority of the free energy has been suggested to arise from non polyelectrolytic forces [57], [72]. All the thermodynamic parameters evaluated from ITC studies performed at three different salt concentrations, viz. 10, 20 and 50 mM are depicted in Table 3.

### Temperature Dependence of the Calorimetric Data: Heat Capacity Changes

**Figure 8:**
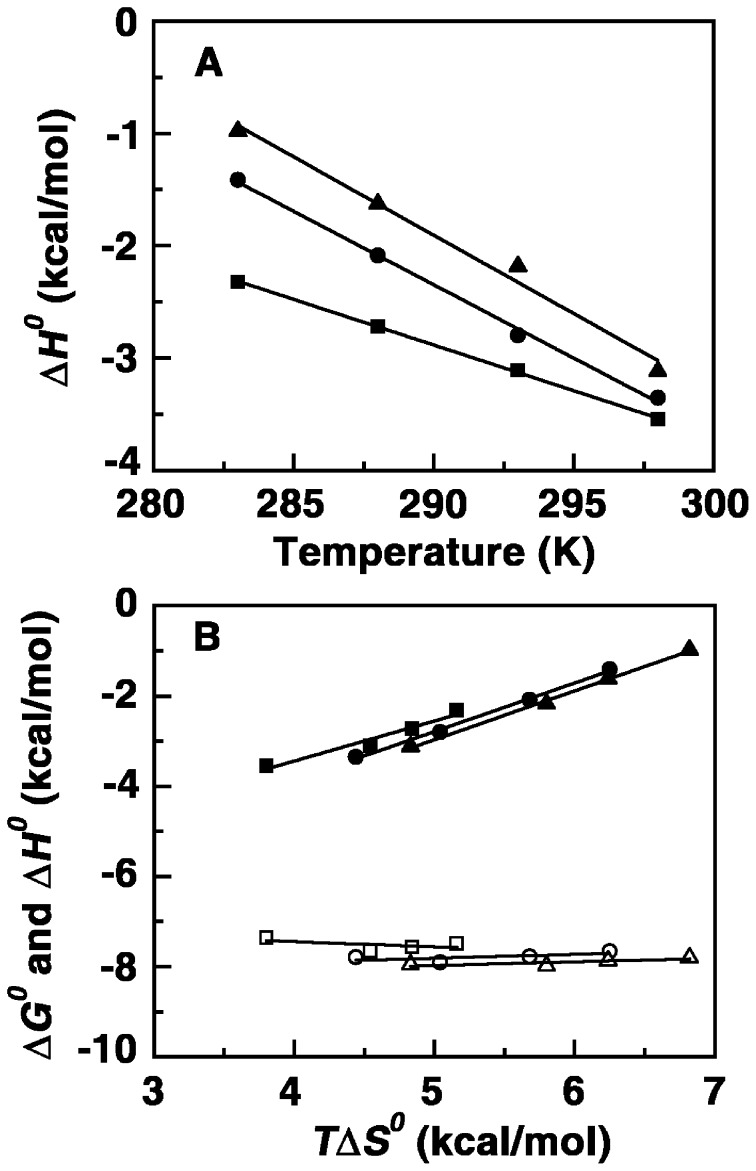
Plots of variation of temperature dependent thermodynamic parameters. (A) Plot of variation of enthalpy of binding (Δ*H^0^*) with temperature for binding of analog B2 (▪), B3 (•) and B4 (▴) to tRNA. (B) Plot of variation of *ΔG^0^* (open symbols) and *ΔH^0^* (closed symbols) versus *TΔ*S*^0^* for the binding of analogs B2 (▪), B3 (•) and B4 (▴) to tRNA, respectively.

To obtain insight about the driving forces of interaction of these analogs with tRNA complex formation was examined as a function of temperature in the range 283.15–298.15 K. Variation of the enthalpy change with temperature can provide information on the heat capacity changes (Δ*C_p_^0^*) of the binding. The observed enthalpy change varied linearly on the experimental temperature for all the alkaloids (Fig. 8A) indicating that there is no measurable shift in the preexisting equilibrium between the conformational states of tRNA in the temperature range studied. Heat capacity provides a link between the structural and energetic data and may describe hydration-dehydration effects that occur during the binding process. Temperature dependence ITC was performed in the range 10° to 25°C and the thermodynamic parameters are elucidated at each temperature. These data are presented in Table 4. Overall, as the temperature increased, the affinity values decreased, the binding enthalpies became more negative with their magnitudes increasing. The negative enthalpy of binding at all temperatures indicated favorable exothermic binding interaction. In all the cases the entropy contribution became more and more unfavorable with increasing temperature whereas the binding enthalpy became more and more favorable at higher temperatures. The energetics of the interaction indicated significant differences in the molecular forces that contribute and control the binding of these analogs to tRNA. Nevertheless, only small changes occurred in the Gibbs energy values. The reaction enthalpy and entropy both of which were strong functions of temperature compensated each other to make the reaction Gibbs energy more or less independent of temperature (Fig. 8B). Such compensation was observed for many biomolecular processes [57], [73], [74] and suggests a significant hydrophobic component to the binding energies. The variation of Δ*H* ^0^ with temperature (Fig. 8A) afforded the Δ*C_p_^0^* values. The slopes of the straight lines gave values of −81.2, −130.6 and −139.4 cal/mol.K, respectively, for the binding of B2, B3 and B4. Negative heat capacity values have been observed for a large variety of small molecules binding to DNA and RNA [13], [47], [57], [68], [71]. Besides, structured water like the water of hydrophobic hydration can be associated with large heat capacity and the release of such water associated with the transfer of the non-polar groups into the interior of the helix can contribute a negative term to the Δ*C_p_^0^*. The Δ*C_p_^0^* values enhanced progressively on going from B2 to B4 probably indicating that the complexation is favoured with increasing chain length. The differences in the Δ*C_p_^0^* values may also indicate differences in the release of structured water consequent to the transfer of nonpolar groups into the interior of the helix. The negative values of Δ*C_p_^0^* in all the cases suggest that the binding is specific and accompanied by burial of non polar surface area [75]–[78].

**Table 4:** Thermodynamic parameters for the association of alkaloids with tRNA from ITC at different temperatures^a^.

Alkaloids	Temperature (K)	*K_a_*/10^5^ M^−1^	Δ*H^0^* (kcal mol^−1^)	*T*Δ*S^0^* (kcalmol^−1^)	Δ*G^0^* (kcal mol^−1^)	Δ*Cp^0^* (cal mol^−1^K^−1^)	Δ*G^o^_hyd_* (kcal mol^−1^)
	283.15	6.08±0.04	−2.32±0.02	5.16	−7.48±0.02		
	288.15	5.43±0.04	−2.72±0.03	4.84	−7.56±0.02		
B2	293.15	4.67±0.03	−3.11±0.04	4.54	−7.65±0.04	−81.2	−6.50
	298.15	2.48±0.02	−3.55±0.06	3.80	−7.35±0.06		
	283.15	8.50±0.05	−1.41±0.02	6.25	−7.66±0.02		
	288.15	7.93±0.05	−2.09±0.03	5.68	−7.77±0.02		
B3	293.15	7.17±0.03	−2.80±0.02	5.04	−7.90±0.02	−130.6	−10.45
	298.15	5.06±0.02	−3.35±0.04	4.44	−7.79±0.04		
	283.15	10.3±0.06	−0.98±0.01	6.82	−7.80±0.01		
	288.15	9.19±0.06	−1.62±0.02	6.24	−7.86±0.02		
B4	293.15	8.20±0.04	−2.18±0.02	5.80	−7.98±0.03	−139.4	−11.15
	298.15	6.55±0.03	−3.12±0.04	4.83	−7.95±0.04		

a All the data in this table are derived from ITC experiments conducted in CP buffer of 10 mM [Na^+^], pH 7.0 and are average of four determinations. *K_a_* and Δ*H^0^* values were determined from ITC profiles fitting to Origin 7.0 software as described in the text. The values of Δ*G^0^* were determined using the equations and Δ*G^0^* = Δ*H^0^-T*Δ*S^0^*. Δ*Cp^0^* is the specific heat capacity and Δ*G^o^_hyd_* is the energy contribution from the hydrophobic transfer step. All the ITC profiles were fit to a model of single binding.

Δ*G^0^_hyd_*, the Gibbs energy contribution for the hydrophobic transfer step for the binding of the alkaloids to tRNA has been calculated from the relationship, described 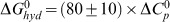 by Record and colleagues [79]. The values of Δ*G^0^_hyd_* for B2-4 binding to tRNA were deduced to be −6.50, −10.45 and −11.15 kcal/mol, respectively (Table 4). These values are well within the range that was observed frequently for DNA and RNA intercalators [22], [47], [57], [77]. It is likely that the thermodynamic components of interaction like the water uptake, water release, etc. may be different and this may be the cause for the slight differences reflected in the Δ*G^0^_hyd_* values.

## Conclusions

The structural aspects of the binding and thermodynamics of interaction of four 9-*O*-substituted berberines with tRNA was studied in comparison with berberine. On selective demethylation at the 9-position of the isoquinoline chromophore the binding affinity drastically reduced but on introduction of the *N*-aryl/arylalkyl amino carbonyl methyl substituent at the 9-position there was a remarkable enhancement in the binding affinity. As the chain length of the substitution increased the affinity enhanced and the highest binding affinity was for B4 (8.20×10^5^ M^−1^) which was about 10 times that of BER under identical conditions. Analysis of the Scatchard plots form absorption and fluorescence titrations revealed that the cooperative binding of BER was propagated in the analogs also. All the 9-*O*-*N*-aryl/arylalkyl amino carbonyl methyl substituted berberine analogs enhanced the thermal stability of tRNA more in comparison to berberine. Ferrocyanide quenching results conclusively proved stronger intercalative geometry for the analogs B2-4 compared to BER, the best being B4 and the data also reflecting weakening of the binding upon selective demethylation at the 9-position of the chromophore. Stronger binding of the analogs was also inferred from circular dichroism studies as the secondary structural changes observed were more pronounced at a lower D/P for the 9-*O*-*N*-aryl/arylalkyl amino carbonyl methyl substituted berberine analogs. The binding of the analogs were predominantly entropy driven with a smaller but favourable enthalpy term, which increased significantly with temperature. Thus, with the introduction of the *N*-aryl/arylalkyl amino carbonyl methyl substituent the entropic contribution to the binding Gibbs energy enhanced suggesting the role of release of tRNA bound water molecules by the side chain. The binding was significantly influenced by salt concentration, but contribution from the non-electrostatic forces to the Gibbs energy of binding was clearly dominant. The negative heat capacity changes in all systems along with significant enthalpy-entropy compensation may be correlated to the involvement of multiple weak non-covalent forces in the complexation process. This study presents a complete spectroscopic and thermodynamic profile of the interaction of 9-*O*-substituted berberine analogs with tRNA that further advances our knowledge on the interaction of small molecules to the tRNA molecule that may prove useful for designing of viable RNA based therapeutic agents.

## Supporting Information

Figure S1 **A plot of change in relative viscosity (η/η_0_) versus D/P (alkaloid/tRNA molar ratio) for BER (o), B2 (▪), B3 (•) and B4 (▴).**
(TIF)

## References

[1] Gesteland RF, Cech TR, Atkins JF (Eds.) (2006) The RNA world. New York, NY: Cold Spring Harbor Laboratory Press.

[2] Ambros V (2004) The functions of animal microRNAs. Nature 431: 350–355. 15372042 10.1038/nature02871

[3] Bartel DP (2004) MicroRNAs: Genomics, biogenesis, mechanism, and function. Cell 116: 281–297. 14744438 10.1016/s0092-8674(04)00045-5

[4] Gait MJ, Karn J (1995) Progress in anti-HIV structure-based drug design. Trends Biotechnol 10: 430–438. 10.1016/S0167-7799(00)88998-2 7546568

[5] Gallego J, Varani G (2001) Targeting RNA with small-molecule drugs: Therapeutic opportunities and chemical challenges. Acc Chem Res 34: 836–843. 11601968 10.1021/ar000118k

[6] Foloppe N, Matassova N, Aboul-Ela F (2006) Towards the discovery of drug-like RNA ligands? Drug Discovery Today 11: 1019–1027. 17055412 10.1016/j.drudis.2006.09.001

[7] Fulle S, Gohlke H (2010) Molecular recognition of RNA: Challenges for modeling interactions and plasticity. J Mol Recognit 23: 220–231. 19941322 10.1002/jmr.1000

[8] Cheong HK, Hwang E, Lee C, Choi BS, Cheong C (2004) Rapid preparation of RNA samples for NMR spectroscopy and X-ray crystallography. Nucleic Acids Res 32: e84. 15199176 10.1093/nar/gnh081 PMC434460

[9] Musselman C, Pitt SW, Gulati K, Foster LL, Andricioaei I, et al (2006) Impact of static and dynamic A-form heterogeneity on the determination of RNA global structural dynamics using NMR residual dipolar couplings. J Biomol NMR 36: 235–249. 17077936 10.1007/s10858-006-9087-9

[10] Nandi R, Debnath D, Maiti M (1990) Interactions of berberine with poly(A) and tRNA. Biochim Biophys Acta 1049: 339–342. 1696505 10.1016/0167-4781(90)90107-d

[11] Islam MM, Sinha R, Suresh Kumar G (2007) RNA binding small molecules: Studies on t-RNA binding by cytotoxic plant alkaloids berberine, palmatine and the comparison to ethidium. Biophys Chem 125: 508–520. 17156912 10.1016/j.bpc.2006.11.001

[12] Islam MM, Pandya P, Roy Chowdhury S, Kumar S, Suresh Kumar G (2008) Binding of DNA-binding alkaloids berberine and palmatine to tRNA and comparison to ethidium: Spectroscopic and molecular modeling studies. J Mol Struct 891: 498–507.

[13] Islam MM, Pandya P, Kumar S, Suresh Kumar G (2009) RNA targeting through binding of small molecules: Studies on t-RNA binding by the cytotoxic protoberberine alkaloid coralyne. Mol BioSyst 5: 244–254. 19225615 10.1039/b816480k

[14] Yadav RC, Suresh Kumar G, Bhadra K, Giri P, Sinha R, et al (2005) Berberine, a strong polyriboadenylic acid binding plant alkaloid: Spectroscopic, viscometric, and thermodynamic study. Bioorg Med Chem 13: 165–174. 15582461 10.1016/j.bmc.2004.09.045

[15] Giri P, Hossain M, Suresh Kumar G (2006) Molecular aspects on the specific interaction of cytotoxic plant alkaloid palmatine to poly(A). Int J Biol Macromol 39: 210–221. 16678250 10.1016/j.ijbiomac.2006.03.026

[16] Giri P, Hossain M, Suresh Kumar G (2006) RNA specific molecules: Cytotoxic plant alkaloid palmatine binds strongly to poly(A). Bioorg Med Chem Lett 16: 2364–2368. 16497501 10.1016/j.bmcl.2006.01.124

[17] Giri P, Suresh Kumar G (2008) Binding of protoberberine alkaloid coralyne with double stranded poly(A): A biophysical study. Mol Biosyst 4: 341–348. 18354787 10.1039/b716356h

[18] Giri P, Suresh Kumar G (2008) Self-structure induction in single stranded poly(A) by small molecules: Studies on DNA intercalators, partial intercalators and groove binding molecules. Arch Biochem Biophys 474: 183–192. 18387354 10.1016/j.abb.2008.03.013

[19] Giri P, Suresh Kumar G (2009) Molecular aspects of small molecules-poly(A) interaction: An approach to RNA based drug design. Curr Med Chem 16: 965–987. 19275606 10.2174/092986709787581932

[20] Giri P, Suresh Kumar G (2010) Isoquinoline alkaloids and their binding with polyadenylic acid: Potential basis of therapeutic action. Mini Rev Med Chem 10: 568–577. 20500148 10.2174/138955710791384009

[21] Islam MM, Basu A, Suresh Kumar G (2011) Binding of 9-*O*-(ω-amino) alkyl ether analogues of the plant alkaloid berberine to poly(A): Insights into self-structure induction. Med Chem Commun 2: 631–637.

[22] Basu A, Jaisankar P, Suresh Kumar G (2012) 9-*O-N*-aryl/arylalkyl amino carbonyl methyl substituted berberine analogues induce self-structure in polyadenylic acid. RSC Adv 2: 7714–7723.

[23] Suresh Kumar G (2012) RNA targeting by small molecules: Binding of protoberberine, benzophenanthridine and aristolochia alkaloids to various RNA structures. J Biosci 37: 539–552. 22750990 10.1007/s12038-012-9217-3

[24] Sinha R, Suresh Kumar G (2009) Interaction of isoquinoline alkaloids with an RNA triplex: Structural and thermodynamic studies of berberine, palmatine, and coralyne binding to poly(U).poly(A)_*_poly(U). J Phys Chem B 113: 13410–13420. 19754095 10.1021/jp9069515

[25] Bhowmik D, Das S, Hossain M, Haq L, Suresh Kumar G (2012) Biophysical characterization of the strong stabilization of the RNA triplex poly(U).poly(A)_*_poly(U) by 9-O-(ω-amino) alkyl ether berberine analogs. PLoS ONE 7: e37939. 22666416 10.1371/journal.pone.0037939 PMC3362543

[26] Hermann T, Westhof E (1998) RNA as a drug target: Chemical, modeling, and evolutionary tools. Curr Opin Biotechnol 9: 66–73. 9503590 10.1016/s0958-1669(98)80086-4

[27] Xavier K, Eder PS, Giordano T (2000) RNA as a drug target: Methods for biophysical characterization and screening. Trends Biotechnol 18: 349–356. 10899816 10.1016/s0167-7799(00)01464-5

[28] Sun Y, Xun K, Wang Y, Chen X (2009) A systematic review of the anticancer properties of berberine, a natural product from Chinese herbs. Anticancer Drugs 20: 757–769. 19704371 10.1097/CAD.0b013e328330d95b

[29] Tang J, Feng Y, Tsao S, Wang N, Curtain R, et al (2009) Berberine and coptidis rhizoma as novel antineoplastic agents: A review of traditional use and biomedical investigations. J Ethnopharmacol 126: 5–17. 19686830 10.1016/j.jep.2009.08.009

[30] Liu JC, Chan P, Chen YJ, Tomlinson B, Hong SH, et al (1999) The antihypertensive effect of the berberine derivative 6-protoberberine in spontaneously hypertensive rats. 59: 283–289. 10.1159/000028331 10575322

[31] Cheuh WH, Lin JY (2012) Protective effect of isoquinoline alkaloid berberine on spontaneous inflammation in the spleen, liver and kidney of non-obese diabetic mice through downregulating gene expression ratios of pro-/anti-inflammatory and Th1/Th2 cytokines. Food Chem 131: 1263–1271.

[32] Janbaz KH, Gilani AH (2000) Studies on preventive and curative effects of berberine on chemical-induced hepatotoxicity in rodents. Fitoterapia 71: 25–33. 11449466 10.1016/s0367-326x(99)00098-2

[33] Shirwaikar A, Shirwaikar A, Rajendran K, Punitha IS (2006) In vitro antioxidant studies on the benzyl tetra isoquinoline alkaloid berberine. 29: 1906–19010. 10.1248/bpb.29.1906 16946507

[34] Peng WH, Lo KL, Lee YH, Hung TH, Lin YC (2007) Berberine produces antidepressant-like effects in the forced swim test and in the tail suspension test in mice. Life Sci 81: 933–938. 17804020 10.1016/j.lfs.2007.08.003

[35] Baird AW, Taylor CT, Brayden DJ (1997) Non-antibiotic anti-diarrhoeal drugs: Factors affecting oral bioavailability of berberine and loperamide in intestinal tissue. Adv Drug Delivery Rev 23: 111–120.

[36] Yan D, Jin C, Xiao XH, Dong XP (2008) Antimicrobial properties of berberines alkaloids in coptis chinensis franch by microcalorimetry. J Biochem Biophys Methods 70: 845–849. 17804078 10.1016/j.jbbm.2007.07.009

[37] Krishnan P, Bastow KF (2000) The 9-position in berberine analogs is an important determinant of DNA topoisomerase II inhibition. Anticancer Drug Des 15: 255–264. 11200501

[38] Zhang WJ, Ou TM, Lu YJ, Huang YY, Wu WB, et al (2007) 9-Substituted berberine derivatives as G-quadruplex stabilizing ligands in telomeric DNA. Bioorg Med Chem 15: 5493–5501. 17574421 10.1016/j.bmc.2007.05.050

[39] Ma Y, Ou TM, Hou JQ, Lu YJ, Tan JH, et al (2008) 9-N-Substituted berberine derivatives: Stabilization of G-quadruplex DNA and down-regulation of oncogene c-myc. Bioorg Med Chem 16: 7582–7591. 18674916 10.1016/j.bmc.2008.07.029

[40] Bodiwala HS, Sabde S, Mitra D, Bhutani KK, Singh IP (2011) Synthesis of 9-substituted derivatives of berberine as anti-HIV agents. Eur J Med Chem 46: 1045–1049. 21295891 10.1016/j.ejmech.2011.01.016

[41] Lo CY, Hsu LC, Chen MS, Lin YJ, Chen LG, et al (2013) Synthesis and anticancer activity of a novel series of 9-O-substituted berberine derivatives: A lipophilic substitute role. Bioorg Med Chem Lett 23: 305–309. 23182088 10.1016/j.bmcl.2012.10.098

[42] Pang JY, Qin Y, Chen WH, Luo GA, Jiang ZH (2005) Synthesis and DNA-binding affinities of monomodified berberines. Bioorg Med Chem 13: 5835–5840. 15993616 10.1016/j.bmc.2005.05.048

[43] Chen WH, Pang JY, Qin Y, Peng Q, Cai Z, et al (2005) Synthesis of linked berberine dimers and their remarkably enhanced DNA-binding affinities. Bioorg Med Chem Lett 15: 2689–2692. 15863343 10.1016/j.bmcl.2004.10.098

[44] Long YH, Bai LP, Qin Y, Pang JY, Chen WH, et al (2006) Spacer length and attaching position-dependent binding of synthesized protoberberine dimers to double-stranded DNA. Bioorg Med Chem 14: 4670–4676. 16563771 10.1016/j.bmc.2006.03.004

[45] Qin Y, Pang JY, Chen WH, Cai Z, Jiang ZH (2006) Synthesis, DNA-binding affinities, and binding mode of berberine dimers. Bioorg Med Chem 14: 25–32. 16169735 10.1016/j.bmc.2005.07.069

[46] Islam MM, Basu A, Hossain M, Sureshkumar G, Hotha S, et al (2011) Enhanced DNA binding of 9-ω-amino alkyl ether analogs from the plant alkaloid berberine. DNA Cell Biol 30: 123–133. 20929398 10.1089/dna.2010.1109

[47] Basu A, Jaisankar P, Suresh Kumar G (2012) Synthesis of novel 9–O-N-aryl/aryl-alkyl amino carbonyl methyl substituted berberine analogs and evaluation of DNA binding aspects. Bioorg Med Chem 20: 2498–2505. 22459209 10.1016/j.bmc.2012.03.006

[48] Sun T, Zhang Y (2008) Pentamidine binds to tRNA through non-specific hydrophobic interactions and inhibits aminoacylation and translation. Nucleic Acids Res 36: 1654–1664. 18263620 10.1093/nar/gkm1180 PMC2275129

[49] Kanakis CD, Nafisi Sh, Rajabi M, Shadaloi A, Tarantilis PA, et al (2009) Structural analysis of DNA and RNA interactions with antioxidant flavanoids. Spectroscopy 23: 29–43.

[50] N'soukpoe-Kossi CN, Ouameur AA, Thomas T, Thomas TJ, Tajmir-Riahi HA (2009) Interaction of tRNA with antitumor polyamine analogues. Biochem Cell Biol 87: 621–630. 19767825 10.1139/o09-036

[51] Ouameur AA, Bourassa P, Tajmir-Riahi HA (2010) Probing tRNA interaction with biogenic polyamines. RNA 16: 1968–1979. 20729276 10.1261/rna.1994310 PMC2941105

[52] Das A, Bhadra K, Suresh Kumar G (2011) Targeting RNA by small molecules: Comparative structural and thermodynamic aspects of aristololactam-β-D-glucoside and daunomycin binding to tRNA^phe^ . PLoS ONE 6: e23186. 21858023 10.1371/journal.pone.0023186 PMC3156712

[53] Hossain M, Kabir A, Suresh Kumar G (2012) Binding of the anticancer alkaloid sanguinarine with tRNA^phe^: Spectroscopic and calorimetric studies. J Biomol Struct Dyn 30: 223–234. 22702734 10.1080/07391102.2012.677774

[54] Iwasa K, Kim HS, Wataya Y, Lee DU (1998) Antimalarial activity and structureactivity relationships of protoberberine alkaloids. Eur J Med Chem 33: 65–69.

[55] Chaires JB, Dattagupta N, Crothers DM (1982) Studies on interaction of anthracycline antibiotics and deoxyribonucleic acid: Equilibrium binding studies on interaction of daunomycin with deoxyribonucleic acid. Biochemistry 21: 3933–3940. 7126524 10.1021/bi00260a005

[56] Bhadra K, Maiti M, Suresh Kumar G (2007) Molecular recognition of DNA by small molecules: AT base pair specific intercalative binding of cytotoxic plant alkaloid palmatine. Biochim Biophys Acta 1770: 1071–1080. 17434677 10.1016/j.bbagen.2007.03.001

[57] Islam MM, Roy Chowdhury S, Suresh Kumar G (2009) Spectroscopic and calorimetric studies on the binding of alkaloids berberine, palmatine and coralyne to double stranded RNA polynucleotides. J Phys Chem B 113: 1210–1224. 19132839 10.1021/jp806597w

[58] McGhee JD, Von Hippel PH (1974) Theoretical aspects of DNA–protein interactions: Cooperative and noncooperative binding of large ligands to a one dimensional homogeneous lattice. J Mol Biol 86: 469–489. 4416620 10.1016/0022-2836(74)90031-x

[59] Bhadra K, Maiti M, Suresh Kumar G (2008) Berberine–DNA complexation: New insights into the cooperative binding and energetic aspects. Biochim Biophys Acta 1780: 1054–1061. 18549823 10.1016/j.bbagen.2008.05.005

[60] Benesi H, Hildebrand J (1949) A spectrophotometric investigation of the interaction of iodine with aromatic hydrocarbons. J Am Chem Soc 71: 2703–2707.

[61] Hoenigman SM, Evans CE (1996) Improved accuracy and precision in the determination of association constants. Anal Chem 68: 3274–3276.

[62] Job P (1928) Formation and stability of inorganic complexes in solution. Ann Chim 9: 113–203.

[63] Huang CY (1982) Determination of binding stoichiometry by the continuous variation method: The Job plot. Methods Enzymol 87: 509–525. 7176926 10.1016/s0076-6879(82)87029-8

[64] Hill ZD, Patrick M (1986) Novel approach to Job's method: An undergraduate experiment. J Chem Educ 63: 162–167.

[65] Lakowicz JR (1983) Principles of fluorescence spectroscopy. New York: Plenum press. pp 257–295.

[66] Garbett NC, Hammond NB, Graves DE (2004) Influence of the amino substituents in the interaction of ethidium bromide with DNA. Biophys J 87: 3974–3981. 15465858 10.1529/biophysj.104.047415 PMC1304907

[67] Giri P, Suresh Kumar G (2008) Spectroscopic and calorimetric studies on the binding of the phototoxic and cytotoxic plant alkaloid sanguinarine with double helical poly(A). Photochem Photobiol A 194: 111–121.

[68] Hossain M, Suresh Kumar G (2009) DNA intercalation of methylene blue and quinacrine: new insights into base and sequence specificity from structural and thermodynamic studies with polynucleotides. Mol Biosyst 5: 1311–1322. 19823747 10.1039/b909563b

[69] Debnath D, Suresh Kumar G, Nandi R, Maiti M (1989) Interaction of berberine chloride with deoxyribonucleic acids: Evidence for base and sequence specificity. Indian J Biochem Bio 26: 201–208. 2628256

[70] Record MT Jr, Anderson CF, Lohman TM (1978) Thermodynamic analysis of ion effects on the binding and conformational equilibria of proteins and nucleic acids: The role of ion association or release, screening, and ion effects on water activity. Q Rev Biophys 11: 103–178. 353875 10.1017/s003358350000202x

[71] Chaires JB (1997) Energetics of drug-DNA interactions. Biopolymers 44: 201–215. 9591476 10.1002/(SICI)1097-0282(1997)44:3<201::AID-BIP2>3.0.CO;2-Z

[72] Bhadra K, Maiti M, Suresh Kumar G (2008) DNA-binding cytotoxic alkaloids: Comparative study of the energetics of binding of berberine, palmatine, and coralyne. DNA Cell Biol 27: 675–685. 18788977 10.1089/dna.2008.0779

[73] Jen-Jacobson L, Engler LE, Jacobson LA (2000) Structural and thermodynamic strategies for site-specific DNA binding proteins. Structure 8: 1015–1023. 11080623 10.1016/s0969-2126(00)00501-3

[74] Chaires JB (2006) A thermodynamic signature for drug-DNA binding mode. Arch Biochem Biophys 453: 26–31. 16730635 10.1016/j.abb.2006.03.027

[75] Murphy FV, Churchill ME (2000) Nonsequence-specific DNA recognition: A structural perspective. Structure 15: R83–R89. 10.1016/s0969-2126(00)00126-x 10801483

[76] Guthrie KM, Parenty ADC, Smith LV, Cronin L, Cooper A (2007) Microcalorimetry of interaction of dihydro-imidazo-phenanthridinium (DIP)-based compounds with duplex DNA. Biophys Chem 126: 117–123. 16750289 10.1016/j.bpc.2006.05.006

[77] Ren J, Jenkins TC, Chaires JB (2000) Energetics of DNA intercalation reactions. Biochemistry 39: 8439–8447. 10913249 10.1021/bi000474a

[78] Buchmueller KL, Bailey SL, Matthews DA, Taherbhai ZT, Register JK, et al (2006) Physical and structural basis for the strong interactions of the –ImPy- central pairing motif in the polyamide f-ImPyIm. Biochemistry 45: 13551–13565. 17087509 10.1021/bi061245c

[79] Ha JH, Spolar RS, Record MT (1989) Role of the hydrophobic effect in stability of site-specific protein-DNA complexes. J Mol Biol 209: 801–816. 2585510 10.1016/0022-2836(89)90608-6

