# Supplementary figures and images for: Binding of the 9-O-N-aryl/arylalkyl Amino Carbonyl Methyl Substituted Berberine Analogs to tRNAphe
Source: PLoS One. 2013 Mar 19;8(3):e58279. doi: 10.1371/journal.pone.0058279 (PMC3602459; doi:10.1371/journal.pone.0058279)

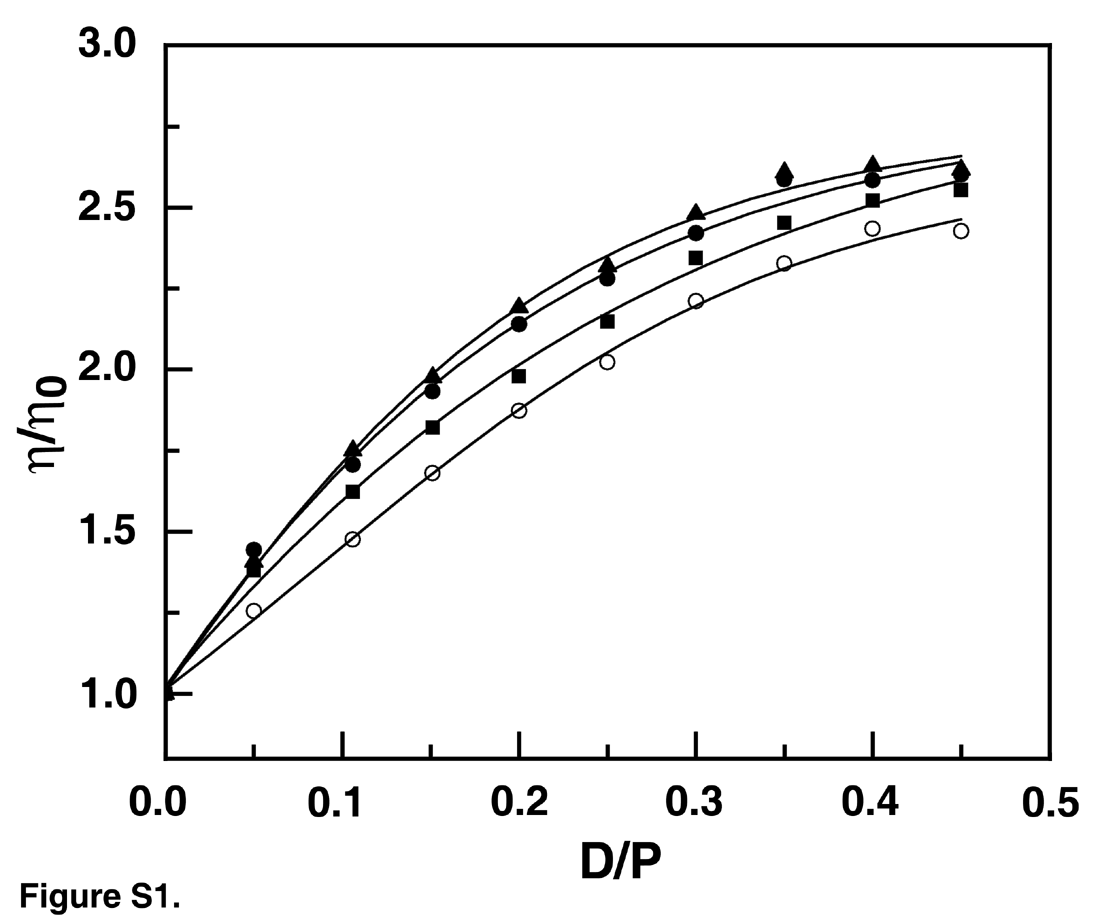

Supplement: Figure S1 — A plot of change in relative viscosity (η/η0) versus D/P (alkaloid/tRNA molar ratio) for BER (o), B2 (▪), B3 (•) and B4 (▴). (TIF) [file pone.0058279.s001.tif]
